# Role of Rotating Cylinder toward Mixed Convection inside a Wavy Heated Cavity via Two-Phase Nanofluid Concept

**DOI:** 10.3390/nano10061138

**Published:** 2020-06-09

**Authors:** Ammar I. Alsabery, Mohammad Ghalambaz, Taher Armaghani, Ali Chamkha, Ishak Hashim, Mohsen Saffari Pour

**Affiliations:** 1 Refrigeration & Air-conditioning Technical Engineering Department, College of Technical Engineering, The Islamic University, Najaf 54001, Iraq; alsabery_a@ukm.edu.my; 2 Department of Mathematical Sciences, Faculty of Science & Technology, Universiti Kebangsaan Malaysia, UKM Bangi 43600, Selangor, Malaysia; ishak_h@ukm.edu.my; 3 Metamaterials for Mechanical, Biomechanical and Multiphysical Applications Research Group, Ton Duc Thang University, Ho Chi Minh City 758307, Vietnam; mohammad.ghalambaz@tdtu.edu.vn; 4 Faculty of Applied Sciences, Ton Duc Thang University, Ho Chi Minh City 758307, Vietnam; 5 Department of Engineering, Mahdishahr Branch, Islamic Azad University, Mahdishahr 75915-35618, Iran; armaghani.taher@yahoo.com; 6 Institute of Research and Development, Duy Tan University, Da Nang 550000, Vietnam; 7 Institute of Theoretical and Applied Research (ITAR), Duy Tan University, Hanoi 100000, Vietnam; 8 Department of Mechanical Engineering, Shahid Bahonar University of Kerman, Kerman 76169-14111, Iran; mohsensp@kth.se; 9 Division of Processes, KTH Royal Institute of Technology, 11428 Stockholm, Sweden

**Keywords:** mixed convection, thermophoresis and Brownian motion, wavy cavity, two-phase nanofluid concept, wavy heater, rotating circular cylinder

## Abstract

The mixed convection two-phase flow and heat transfer of nanofluids were addressed within a wavy wall enclosure containing a solid rotating cylinder. The annulus area between the cylinder and the enclosure was filled with water-alumina nanofluid. Buongiorno’s model was applied to assess the local distribution of nanoparticles in the host fluid. The governing equations for the mass conservation of nanofluid, nanoparticles, and energy conservation in the nanofluid and the rotating cylinder were carried out and converted to a non-dimensional pattern. The finite element technique was utilized for solving the equations numerically. The influence of the undulations, Richardson number, the volume fraction of nanoparticles, rotation direction, and the size of the rotating cylinder were examined on the streamlines, heat transfer rate, and the distribution of nanoparticles. The Brownian motion and thermophoresis forces induced a notable distribution of nanoparticles in the enclosure. The best heat transfer rate was observed for 3% volume fraction of alumina nanoparticles. The optimum number of undulations for the best heat transfer rate depends on the rotation direction of the cylinder. In the case of counterclockwise rotation of the cylinder, a single undulation leads to the best heat transfer rate for nanoparticles volume fraction about 3%. The increase of undulations number traps more nanoparticles near the wavy surface.

## 1. Introduction

Natural convection and heat transfer mechanisms in the annulus spaces have been the topic of many pioneer investigations due to its essential engineering applications. Such tools into enclosed spaces give strong non-linear behavior due to the effective coupling between the flow and heat equation. Shu et al. [1] modeled the free convection heat transfer within a square outer cylinder and a circular central cylinder. The authors assumed an isothermal hot temperature at the cylinder and cold isothermal temperature at the enclosure boundaries. Shu et al. [1] probed the influence of the location of a cylinder toward the measure of the heat transfer and fluid circulation. They found that the top portion within the enclosure boundaries and cylinder has a significant use in the formation of the natural convection plume. In another study, Shu and Zhu [2] investigated the outcome of the aspect ratio (the ratio of cavity size/cylinder diameter) at the natural convection heat transfer, and they reported that both Rayleigh number and aspect ratio are critical to the flow patterns and thermal fields. The natural convection heat transfer in the annulus space among a cylinder and a square was also investigated by Ding et al. [3], Angeli et al. [4], and Alsabery et al. [5].

The present study involves the conjugate heat transfer, rotating cylinder, wavy wall enclosures and nanofluids. Hence, the literature works related to these topics are explored here. The natural convection heat transfer in enclosures containing solid thermal conductive blocks has been studied in some recent studies. The presence of a solid block contributes to the heat transfer inside the enclosure while it affects the fluid circulation. This type of problems is classified as conjugate heat transfer as the liquid and solid are in thermal interaction. Kuznetsov and Sheremet [6] modeled the free convection within an air-filled enclosure containing a solid block. The block enclosed a heat source. Jami et al. [7] examined the heat transfer in a cavity filled including solid cylinder at various locations. These authors reported that the area of the cylinder is the critical parameter testing the flow circulation and heat transfer.

Sheremet [8] examined the mechanism of heat transfer into a cylindrical cavity holding a solid heated block toward the bottom portion. The results show that a cylindrical cavity can be of essential advantages for the cooling of electronic components. Butler et al. [9] experimentally inspected the conjugate natural convection mechanism and heat transfer over a cylinder enclosed inside an air-filled cubic enclosure. The left and right vertical sidewalls of the cavity were at temperature difference while the other walls were insulated. The observations indicate that the presence of the cylinder could interfere with the structure of natural convection flow circulation in the enclosure. Various aspect of conjugate heat transfer toward a cavity such as radiation [10], mixed convection [11], and turbulent effects [12] have been addressed during past years.

The presence of a moving or rotating object changes the natural convection flow to the mixed convection. The moving objects have found numerous applications in engineering designs. For instance, a shaft of turbine or pump enclosed in a shell, rotation of gears in a casing, vibration of a thermal fin in an enclosed space, and fluid bearings with a slow rotation, and the receiver of a solar collector are just a few examples. Costa and Raimundo [13] displayed the combined convection and heat transfer mechanisms within a square hollow holding a rotating solid cylinder. The vertical surfaces of the cavity occurred with cold and hot temperatures, and the top and bottom surfaces remained insulated. The cylinder was rotating at a certain angular velocity and contributed into the flow circulation and heat transfer. The results show that the cylinder’s size induces a notable impact on the fluid circulation and transfer since it confines the liquid area for fluid flow inside the cavity. However, the angular velocity of the cylinder is another important parameter. The cylinder rotation could improve the rate of the heat transfer at the enclosure aspect ratio (the cylinder radius to the cavity size) is large. However, in the case of small aspect ratio (small cylinder), the rotation of the cylinder could deteriorate the overall heat transfer inside the cavity. Wang et al. [14] studied the mixed convection technique and entropy generation of a rotating hollow cylinder within a square hollow. They found that the increase in rotation velocity boosts the total entropy generation. Many aspects of mixed convection and heat transfer mechanisms within the geometry of a rotating cylinder in a square cavity have been addressed. For example, the appearance of a porous medium layer [15], the location of rotating cylinder [16], rotation speed [17,18], and two rotating cylinders [19] have been explored in the literature.

Enclosures with wavy (curved) walls have numerous practical applications in solar systems, heat exchangers, and reactors. Hence, the convective heat transfer in enclosures with wavy walls has been investigated in numerous recent studies. Adjlout et al. [20] questioned the natural convection flow, including heat transfer in a tilted wavy wall cavity. They explored the influence of the number of the undulations of the wall on the heat transfer. The undulation of hot wall reduced the heat transfer rate for an inclination angle greater than 75°. Bhardwaj et al. [21] analyzed the natural convection heat transfer toward a porous-filled triangular cavity including a curved cold wall. They found that undulations on the cold wall improve the heat transfer rate. Considering the wavy wall forms, the entropy generation [22,23], non-uniform heating [24], partially heated wall [25,26], and micropolar fluids [27] have been investigated in recent years.

Using nanotechnology in the improvement of heat transfer, by scattering nanosized solids with high conductivity such as metal, metal oxide, and carbon single- and multi-layer tube in such base fluids as water, oil, and other standard coolants has been investigated in the past decades [28]. Using nanofluids is considered as a proper choice in fluid-based cooling. Heat transfer of nanofluids has, therefore, a wide variety of applications such as cooling electronic devices and chips. Considering the considerable bulk of calculations and hence the generated heat, CPU and electronic chips need fluids to be cooled, so nanofluids are cutting edge technology in this regard [29,30]. Therefore, modeling the nanofluid heat transfer is a significant problem in this field. Accurate modeling of the motion of nanoparticles into the base liquid has complexities. One of the recommended models for simulating the heat transfer of nanofluids is Buongiorno’s two-phase approach. In this model, two bold movements of nanoparticles, due to changes of temperature and volume fraction distribution, are analyzed as valid parameters, along with other aspects of the classic equations of survival [31]. In addition, in this model, the non-uniform distribution of nanoparticles is studied. On the other hand, the migration of particles has an important role in heat transfer of nanofluid. As shown by Buongiorno [31], two migration term called Brownian movement and thermophoresis effects have a significant role in heat transfer of nanofluids. Many researchers have used Buongiorno’s two-phase model to simulate the flow and heat transfer of nanofluids [32,33,34,35,36,37,38,39].

The accordance of mixed convection heat transfer due to the rigid rotating body is one of the challenges of nanofluids heat transfer, about which few articles have been published. In this study, by using Buongiorno’s model, mixed convection of a nanofluid in the cavity, along with warm corrugated wall and a rigid rotating body, was analyzed. To the authors’ knowledge and according to the literature mentioned above, the current study is unique and initiative. Moreover, when a nanofluid is synthesized, it is placed into the application to see how the synthesized nanofluid could improve the heat transfer. Various aspects, such as dynamic viscosity and migration of nanoparticles, would participate in the heat transfer behavior of a nanofluid.

## 2. Mathematical Formulation

**Figure 1:**
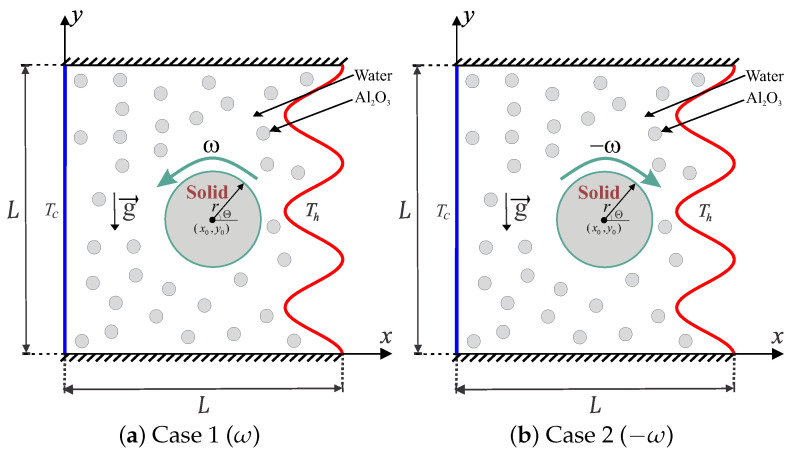
Schematic diagram of the physical model together with the coordinate system for: (**a**) Case 1, counterclockwise (CCW); and (**b**) Case 2, clockwise (CW).

The mixed convective heat transfers into a wavy-walled cavity by length *L* and continues as a rotating solid cylinder inside the center with radius *r*, as outlined in Figure 1. The left vertical surface preserves a fixed cold temperature ($T_{c}$) while the right wavy surface remains at a higher isothermal temperature ($T_{h}$). The bottom and top surfaces are maintained adiabatic. All the edges of the examined domain are expected to be impermeable, the fluid inside the hollow is a water-based nanofluid having Al_2_O_3_ nanoparticles, and the Boussinesq approximation remains applicable. In the present laminar flow study, the surface effects on the concentration distribution of nanoparticles are neglected. Examining the assumptions mentioned above, the continuity, momentum, and energy equations regarding the laminar and steady convection are as follows:

(1) $$\begin{array}{c} \nabla \cdot v=0, \end{array}$$

(2) $$\begin{array}{c} \rho_{nf}v\cdot \nabla v=-\nabla p+\nabla \cdot \left(\mu_{nf}\nabla v\right)+{\left(\rho \beta \right)}_{nf}\left(T-T_{c}\right)\vec{g}, \end{array}$$

(3) $$\begin{array}{c} {\left(\rho C_{p}\right)}_{nf}v\cdot \nabla T=\nabla \cdot \left(k_{nf}\nabla T\right)-C_{p,p}J_{p}\cdot \nabla T, \end{array}$$

(4) $$\begin{array}{c} v\cdot \nabla \varphi =-\frac{1}{\rho_{p}}\nabla \cdot J_{p}, \end{array}$$

Since the inner cylinder denotes a moving mass block including an associated force, the energy equation up the solid cylinder is:

(5) $${\left(\rho C_{p}\right)}_{s}v_{s}\cdot \nabla T=\nabla \cdot \left(k_{s}\nabla T\right),$$

where v is the velocity vector, $v_{s}=r\times \omega$ is the vector velocity over the solid cylinder surface, $\vec{g}$ means the gravitational acceleration vector, φ denotes the local nanoparticles volume fraction, and $J_{p}$ is the mass flux of nanoparticles. According to the two-phase nanofluid model, nanoparticles mass flux can be formulated as:

(6) $$\begin{array}{c} J_{p}=J_{p,B}+J_{p,T}, \end{array}$$

(7) $$\begin{array}{c} J_{p,B}=-\rho_{p}D_{B}\nabla \varphi ,\;D_{B}=\frac{k_{b}T}{3\pi \mu_{f}d_{p}}, \end{array}$$

(8) $$\begin{array}{c} J_{p,T}=-\rho_{p}D_{T}\frac{\nabla T}{T},\;D_{T}=0.26\frac{k_{f}}{2k_{f}+k_{p}}\frac{\mu_{f}}{\rho_{f}T}\varphi . \end{array}$$

The thermophysical properties of nanofluids including effective thermal diffusivity, heat capacitance, thermal expansion coefficient, and effective density are addressed as, respectively,

(9) $$\begin{array}{ccc} \alpha_{nf} & = & \frac{k_{nf}}{{\left(\rho C_{p}\right)}_{nf}}, \end{array}$$

(10) $$\begin{array}{ccc} {\left(\rho C_{p}\right)}_{nf} & = & \left(1-\varphi \right){\left(\rho C_{p}\right)}_{f}+\varphi {\left(\rho C_{p}\right)}_{p}, \end{array}$$

(11) $$\begin{array}{ccc} {\left(\rho \beta \right)}_{nf} & = & \left(1-\varphi \right){\left(\rho \beta \right)}_{f}+\varphi {\left(\rho \beta \right)}_{p}, \end{array}$$

(12) $$\begin{array}{ccc} \rho_{nf} & = & \left(1-\varphi \right)\rho_{f}+\varphi \rho_{p}. \end{array}$$

The thermal conductivity ratio of Al_2_O_3_-water nanofluids calculated by the Corcione model [40] is:

(13) knfkf=1+4.4ReB0.4Pr0.66TTfr10kpkf0.03φ0.66,

where ReB is shown as [40]:

(14) ReB=ρfuBdpμf,uB=2kbTπμfdp2.

Here, $k_{b}=1.380648\times 10^{-23}\left(J/K\right)$ is the Boltzmann constant, $l_{f}=0.17$ nm is the mean path of fluid particles, and $d_{f}$ is the molecular diameter of water given by Corcione [40] as:

(15) $$d_{f}=\frac{6M}{N^{*}\pi \rho_{f}},$$

where *M* denotes the molecular weight of the base liquid, $N^{*}$ means the Avogadro number, and $\rho_{f}$ is the density of the base liquid toward the regular temperature (310 K).

We propose the following non-dimensional variables:

(16) $$\begin{array}{cc} X & =\frac{x}{L},\;\;Y=\frac{y}{L},\;\;V=\frac{vL}{\nu_{f}},\;\;P=\frac{pL^{2}}{\rho_{nf}\nu_{f}^{2}},\;\;\varphi^{*}=\frac{\varphi }{\phi },\\ D_{B}^{*} & =\frac{D_{B}}{D_{B0}},\;\;D_{T}^{*}=\frac{D_{T}}{D_{T0}},\;\;\delta =\frac{T_{h}-T_{c}}{T_{c}},\\ \theta & =\frac{T-T_{c}}{T_{h}-T_{c}},\;\;\theta_{s}=\frac{T_{s}-T_{c}}{T_{h}-T_{c}},\;\;R=\frac{r}{L},\;\;\Omega =\frac{\omega L^{2}}{\alpha_{f}}. \end{array}$$

Applying the variables mentioned above, the following dimensionless governing equations are derived:

(17) $$\begin{array}{c} \nabla \cdot V=0, \end{array}$$

(18) $$\begin{array}{c} V\cdot \nabla V=-\nabla P+\frac{\rho_{f}}{\rho_{nf}}\frac{\mu_{nf}}{\mu_{f}}\frac{1}{Re}\nabla^{2}V+\frac{{\left(\rho \beta \right)}_{nf}}{\rho_{nf}\beta_{f}}Ri\cdot \theta , \end{array}$$

(19) $$\begin{array}{cc} V\cdot \nabla \theta & =\frac{{\left(\rho C_{p}\right)}_{f}}{{\left(\rho C_{p}\right)}_{nf}}\frac{k_{nf}}{k_{f}}\frac{1}{Re\cdot \mathrm{Pr}}\nabla^{2}\theta +\frac{{\left(\rho C_{p}\right)}_{f}}{{\left(\rho C_{p}\right)}_{nf}}\frac{D_{B}^{*}}{Re\cdot \mathrm{Pr}\cdot Le}\nabla \varphi^{*}\cdot \nabla \theta \\ \; & +\frac{{\left(\rho C_{p}\right)}_{f}}{{\left(\rho C_{p}\right)}_{nf}}\frac{D_{T}^{*}}{Re\cdot \mathrm{Pr}\cdot Le\cdot N_{BT}}\frac{\nabla \theta \cdot \nabla \theta }{1+\delta \theta }, \end{array}$$

(20) $$\begin{array}{c} V\cdot \nabla \varphi^{*}=\frac{D_{B}^{*}}{Re\cdot Sc}\nabla^{2}\varphi^{*}+\frac{D_{T}^{*}}{Re\cdot Sc\cdot N_{BT}}\cdot \frac{\nabla^{2}\theta }{1+\delta \theta }, \end{array}$$

(21) $$\begin{array}{c} V_{s}\cdot \nabla \theta =\frac{{\left(\rho C_{p}\right)}_{nf}}{{\left(\rho C_{p}\right)}_{s}}\frac{k_{s}}{k_{nf}}\nabla^{2}\theta , \end{array}$$

where V shows the dimensionless vector of velocity ($U_{0},V_{0}$), $D_{B0}=\frac{k_{b}T_{c}}{3\pi \mu_{f}d_{p}}$ is the reference coefficient of Brownian diffusion $\left(D_{B}^{*}=\frac{D_{B}}{D_{B0}}\right)$, $D_{T0}=0.26\frac{k_{f}}{2k_{f}+k_{p}}\frac{\mu_{f}}{\rho_{f}T_{c}}\phi$ is the reference coefficient of thermophoretic diffusion $\left(D_{T}^{*}=\frac{D_{T}}{D_{T0}}\right)$, $Sc=\frac{\nu_{f}}{D_{B0}}$ is Schmidt number, $N_{BT}=\frac{\phi D_{B0}T_{c}}{D_{T0}\left(T_{h}-T_{c}\right)}$ is the parameter of diffusivity ratio (Brownian diffusivity/thermophoretic diffusivity), $Le=\frac{k_{f}}{{\left(\rho C_{p}\right)}_{f}\phi D_{B0}}$ is the Lewis number, $Re=\frac{U_{0}L}{\nu_{f}}$ is Reynolds number, $Ri=\frac{Gr}{{Re}^{2}}$ is Richardson number, and $\mathrm{Pr}=\nu_{f}/\alpha_{f}$ is the Prandtl number for the base fluid. The dimensionless boundary conditions of Equations (17)–(21) are:

$$\begin{array}{c} \mathrm{On}\;\mathrm{the}\;\mathrm{adiabatic}\;\mathrm{top}\;\mathrm{horizontal}\;\mathrm{wall}: \end{array}$$

(22) $$\begin{array}{c} U=V=0,\;\;\frac{\partial \varphi^{*}}{\partial n}=0,\;\frac{\partial \theta }{\partial n}=0, \end{array}$$

Onthecoldleftverticalwall:

(23) $$\begin{array}{c} U=V=0,\;\;\frac{\partial \varphi^{*}}{\partial n}=-\frac{D_{T}^{*}}{D_{B}^{*}}\cdot \frac{1}{N_{BT}}\cdot \frac{1}{1+\delta \theta }\frac{\partial \theta }{\partial n},\;\theta =0, \end{array}$$

Ontheheatedrightwavywall:A(1−cos(2NπX)),0≤Y≤1

(24) $$\begin{array}{c} U=V=0,\;\;\frac{\partial \varphi^{*}}{\partial n}=-\frac{D_{T}^{*}}{D_{B}^{*}}\cdot \frac{1}{N_{BT}}\cdot \frac{1}{1+\delta \theta }\frac{\partial \theta }{\partial n},\;\theta =1, \end{array}$$

Ontheadiabaticbottomhorizontalwall:

(25) $$\begin{array}{c} U=V=0,\;\;\frac{\partial \varphi^{*}}{\partial n}=0,\;\frac{\partial \theta }{\partial n}=0, \end{array}$$

(26) $$\theta =\theta_{s},\;\;\mathrm{at}\;\mathrm{the}\;\mathrm{outer}\;\mathrm{solid}\;\mathrm{cylinder}\;\mathrm{surface},$$

(27) $$\begin{array}{cc} U=- & \Omega \left(Y-Y_{0}\right),\;V=\Omega \left(X-X_{0}\right),\\ \; & \frac{\partial \varphi^{*}}{\partial n}=-\frac{D_{T}^{*}}{D_{B}^{*}}\cdot \frac{1}{N_{BT}}\cdot \frac{1}{1+\delta \theta }\frac{\partial \theta }{\partial n},\;\;\frac{\partial \theta }{\partial n}=K_{r}\frac{\partial \theta_{s}}{\partial n}, \end{array}$$

where $K_{r}=k_{s}/k_{nf}$ is the thermal conductivity ratio upper the surface of the rotating conductive cylinder.

The boundary conditions for nanoparticles are dictated from the physics of nanofluid, in which the nanoparticles cannot penetrate the surface of the enclosure. The hydraulic boundary conditions are prescribed from the fact that the velocity of a fluid and the adjacent surface should be identical. The thermal boundary conditions simulate the heat transfer of a rotating shaft in a housing. The cavity is cooled by the side walls while the other side is subject to a process system of hot temperature.

Regarding the nanoparticles, we employed Buongiorno’s mathematical model to investigate the concentration distribution of nanoparticles in the host fluid when the liquid is exposed to temperature gradients. The size of variation in the size of the nanoparticles can affect the concentration distribution of nanoparticles. As we used the average size of nanoparticles in a sample, the produced concentration distribution would also show the average of possible concentration distributions. Such outcomes could be adequate for most of the engineering designs.

The local Nusselt number ($Nu_{nf}$) evaluated at the hot wavy wall of the cavity is represented by:

(28) $$Nu_{nf}=-\frac{k_{nf}}{k_{f}}{\left(\frac{\partial \theta }{\partial W}\right)}_{W}.$$

In addition, we can define the interface local Nusselt number ($Nu_{i}$) evaluated at the interface wall between the rotating conductive cylinder and the wavy-walled cavity as follows:

(29) $$Nu_{i}=-{\left(\frac{\partial \theta_{s}}{\partial S}\right)}_{S}.$$

where *W* and *S* represent the total length of the wavy heater and the interface wall around the rotating solid cylinder, respectively. The average Nusselt number evaluated at the hot wavy wall is defined as follows:

(30) $$\begin{array}{c} {\bar{Nu}}_{nf}=\int_{0}^{W}Nu_{nf}\;dW. \end{array}$$

## 3. Numerical Method and Validation

The dimensionless form of the governing equations in Equations (17)–(21) controlled by dimensionless boundary conditions in Equations (22)–(27) were solved by the Galerkin weighted residual finite element method. First, we transferred the momentum equations in Equation (18) to the Cartesian *X* and *Y* coordinates as follows:

The momentum equation in the *X*-direction:

(31) $$\begin{array}{c} U\frac{\partial U}{\partial X}+V\frac{\partial U}{\partial Y}=-\frac{\partial P}{\partial X}+\frac{\rho_{f}}{\rho_{nf}}\frac{\mu_{nf}}{\mu_{f}}\frac{1}{Re}\left(\frac{\partial^{2}U}{\partial X^{2}}+\frac{\partial^{2}U}{\partial Y^{2}}\right). \end{array}$$

The momentum equation in the *Y*-direction:

(32) $$\begin{array}{cc} & U\frac{\partial U}{\partial X}+V\frac{\partial U}{\partial Y}=-\frac{\partial P}{\partial Y}+\frac{\rho_{f}}{\rho_{nf}}\frac{\mu_{nf}}{\mu_{f}}\frac{1}{Re}\left(\frac{\partial^{2}V}{\partial X^{2}}+\frac{\partial^{2}V}{\partial Y^{2}}\right)+\frac{{\left(\rho \beta \right)}_{nf}}{\rho_{nf}\beta_{f}}Ri\;\theta \end{array}$$

The Finite Element Method (FEM) was employed to solve the governing equations. Applying the FEM to the momentum in Equations (31) and (32) leads to the following process:

Primary, we applied the penalty FEM by excluding the pressure (*P*) including a penalty parameter (λ) as:

(33) $$\begin{array}{c} P=-\lambda \left(\frac{\partial U}{\partial X}+\frac{\partial V}{\partial Y}\right). \end{array}$$

This led to the following momentum equations:

(34) $$\begin{array}{ccc} U\frac{\partial U}{\partial X}+V\frac{\partial U}{\partial Y} & = & \frac{\partial \lambda }{\partial X}\left(\frac{\partial U}{\partial X}+\frac{\partial V}{\partial Y}\right)+\frac{\rho_{f}}{\rho_{nf}}\frac{\mu_{nf}}{\mu_{f}}\frac{1}{Re}\left(\frac{\partial^{2}U}{\partial X^{2}}+\frac{\partial^{2}U}{\partial Y^{2}}\right),\\ U\frac{\partial V}{\partial X}+V\frac{\partial V}{\partial Y} & = & \frac{\partial \lambda }{\partial Y}\left(\frac{\partial U}{\partial X}+\frac{\partial V}{\partial Y}\right)+\frac{\rho_{f}}{\rho_{nf}}\frac{\mu_{nf}}{\mu_{f}}\frac{1}{Re}\left(\frac{\partial^{2}V}{\partial X^{2}}+\frac{\partial^{2}V}{\partial Y^{2}}\right)+\frac{{\left(\rho \beta \right)}_{nf}}{\rho_{nf}\beta_{f}}Ri\;\theta . \end{array}$$

The weak (or weighted-integral) formulation of the momentum equations was obtained by multiplying the equation with an internal domain (Φ) and integrating it over the computational domain. The following weak formulations were then obtained:

(35) $$\begin{array}{c} \int_{\Omega }\left(\Phi_{i}U^{k}\frac{\partial U^{k}}{\partial X}+\Phi_{i}V^{k}\frac{\partial U^{k}}{\partial Y}\right)dXdY=\lambda \int_{\Omega }\frac{\partial \Phi_{i}}{\partial X}\left(\frac{\partial U^{k}}{\partial X}+\frac{\partial V^{k}}{\partial Y}\right)dXdY\\ \;+\frac{\rho_{f}}{\rho_{nf}}\frac{\mu_{nf}}{\mu_{f}}\frac{1}{Re}\int_{\Omega }\Phi_{i}\left(\frac{\partial^{2}U^{k}}{\partial X^{2}}+\frac{\partial^{2}U^{k}}{\partial Y^{2}}\right)dXdY, \end{array}$$

(36) $$\begin{array}{c} \int_{\Omega }\left(\Phi_{i}V^{k}\frac{\partial V^{k}}{\partial X}+\Phi_{i}V^{k}\frac{\partial V^{k}}{\partial Y}\right)dXdY=\lambda \int_{\Omega }\frac{\partial \Phi_{i}}{\partial Y}\left(\frac{\partial U^{k}}{\partial X}+\frac{\partial V^{k}}{\partial Y}\right)dXdY\\ \;+\frac{\rho_{f}}{\rho_{nf}}\frac{\mu_{nf}}{\mu_{f}}\frac{1}{Re}\int_{\Omega }\Phi_{i}\left(\frac{\partial^{2}V^{k}}{\partial X^{2}}+\frac{\partial^{2}V^{k}}{\partial Y^{2}}\right)dXdY+\frac{{\left(\rho \beta \right)}_{nf}}{\rho_{nf}\beta_{f}}Ri\int_{\Omega }\Phi_{i}\theta^{k}dXdY, \end{array}$$

where the superscript *k* is the relative index. The interpolation functions including any of the velocity distribution, temperature, and the nanoparticle distribution are approximated by employing a basis set ${\left\{\Phi_{j}\right\}}_{j=1}^{N}$ as,

(37) $$\begin{array}{cc} & V\approx \sum_{j=1}^{N}V_{j}\Phi_{j}\left(X,Y\right),\;\theta \approx \sum_{j=1}^{N}\theta_{j}\Phi_{j}\left(X,Y\right),\;\varphi^{*}\approx \sum_{j=1}^{N}\varphi_{j}^{*}\Phi_{j}\left(X,Y\right). \end{array}$$

Then, the residual form of equations was computed by integrating the weak form of equations over a discrete domain:

(38) $$\begin{array}{ccc} R{\left(1\right)}_{i} & = & \sum_{j=1}^{m}U_{j}\int_{\Omega }\left[\left(\sum_{j=1}^{m}U_{j}\Phi_{j}\right)\frac{\partial \Phi_{j}}{\partial X}+\left(\sum_{j=1}^{m}V_{j}\Phi_{j}\right)\frac{\partial \Phi_{j}}{\partial Y}\right]\Phi_{i}dXdY\\ \; & \; & +\lambda \left[\sum_{j=1}^{m}U_{j}\int_{\Omega }\frac{\partial \Phi_{i}}{\partial X}\frac{\partial \Phi_{j}}{\partial X}dXdY+\sum_{j=1}^{m}V_{j}\int_{\Omega }\frac{\partial \Phi_{i}}{\partial X}\frac{\partial \Phi_{j}}{\partial Y}dXdY\right]\\ \; & \; & +\frac{\rho_{f}}{\rho_{nf}}\frac{\mu_{nf}}{\mu_{f}}\frac{1}{Re}\sum_{j=1}^{m}U_{j}\int_{\Omega }\left[\frac{\partial \Phi_{i}}{\partial X}\frac{\partial \Phi_{j}}{\partial X}+\frac{\partial \Phi_{i}}{\partial Y}\frac{\partial \Phi_{j}}{\partial Y}\right]dXdY, \end{array}$$

(39) $$\begin{array}{ccc} R{\left(2\right)}_{i} & = & \sum_{j=1}^{m}V_{j}\int_{\Omega }\left[\left(\sum_{j=1}^{m}U_{j}\Phi_{j}\right)\frac{\partial \Phi_{j}}{\partial X}+\left(\sum_{j=1}^{m}V_{j}\Phi_{j}\right)\frac{\partial \Phi_{j}}{\partial Y}\right]\Phi_{i}dXdY\\ \; & \; & +\lambda \left[\sum_{j=1}^{m}U_{j}\int_{\Omega }\frac{\partial \Phi_{i}}{\partial Y}\frac{\partial \Phi_{j}}{\partial X}dXdY+\sum_{j=1}^{m}V_{j}\int_{\Omega }\frac{\partial \Phi_{i}}{\partial Y}\frac{\partial \Phi_{j}}{\partial Y}dXdY\right]\\ \; & \; & +\frac{\rho_{f}}{\rho_{nf}}\frac{\mu_{nf}}{\mu_{f}}\frac{1}{Re}\sum_{j=1}^{m}V_{j}\int_{\Omega }\left[\frac{\partial \Phi_{i}}{\partial X}\frac{\partial \Phi_{j}}{\partial X}+\frac{\partial \Phi_{i}}{\partial Y}\frac{\partial \Phi_{j}}{\partial Y}\right]dXdY\\ \; & \; & +\frac{{\left(\rho \beta \right)}_{nf}}{\rho_{nf}\beta_{f}}Ri\int_{\Omega }\left(\sum_{j=1}^{m}\theta_{j}\Phi_{j}\right)\Phi_{i}dXdY, \end{array}$$

where the relative index is denoted by the superscript *k* and subscripts of *i* and *j* represent the residual and node number, respectively. Here, *m* shows the iteration number. The integrals were performed by second-order Gaussian quadrature. The Newton–Raphson iteration algorithm was applied to iteratively solve the residual equations with the following stopping condition for every field variable:

(40) $$\begin{array}{c} \left|\frac{\Gamma^{m+1}-\Gamma^{m}}{\Gamma^{m+1}}\right|\leq \eta . \end{array}$$

where *m* represents the iteration number and η is the convergence criterion.

**Figure 2:**
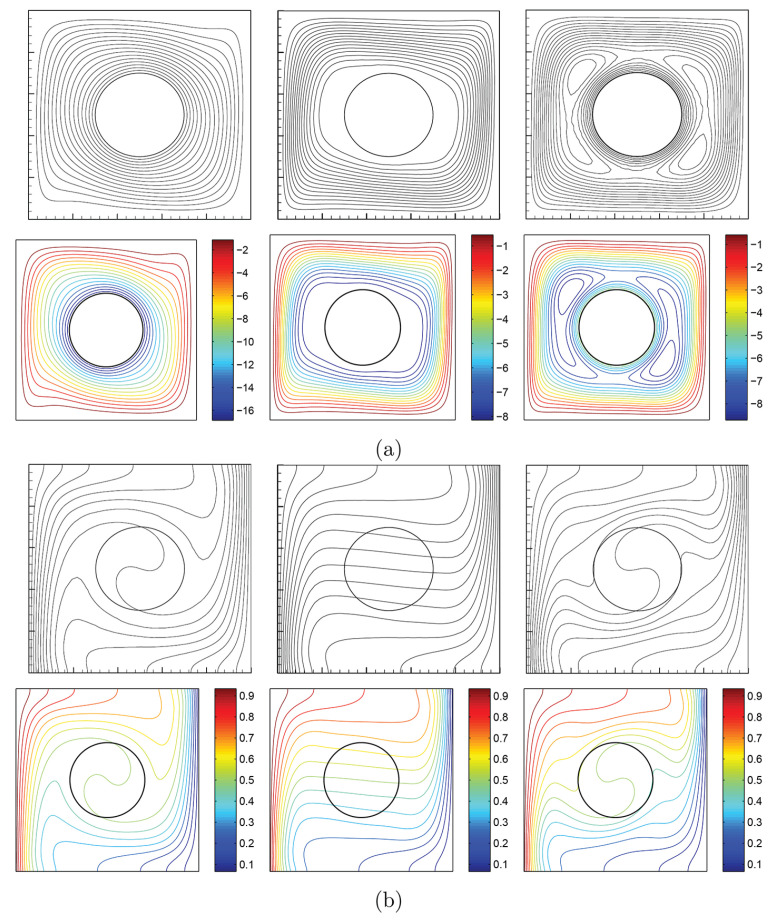
Comparisons between the results of (**top**) Costa and Raimundo [13] and (**bottom**) the present work for Ω=−500 (**left**), Ω=0 (**middle**), and Ω=500 (**right**) of streamlines (**a**) and isotherms (**b**) at $Ra=10^{5}$, N=0, $K_{r}=1$, R=0.2, and Pr=0.7.

**Figure 3:**
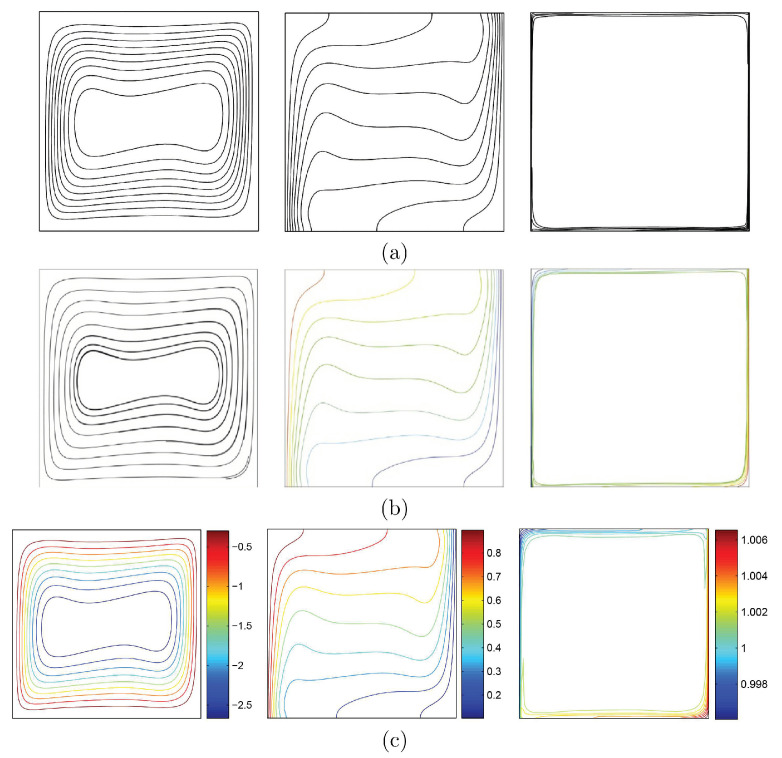
Validations of (**left**) streamlines, (**middle**) isotherms, and (**right**) nanoparticle distribution for (**a**) Corcione et al. [41], (**b**) Wang et al. [42], and (**c**) the present study at $Ra=3.37\times 10^{5}$, ϕ=0.04, N=0, and R=0.

**Figure 4:**
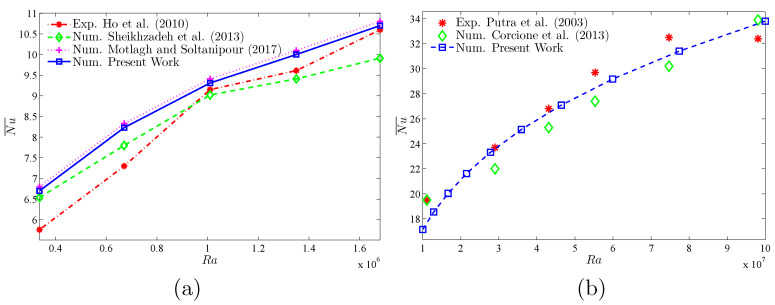
Comparisons of the average Nusselt number of the current numerical work with: (**a**) the experimental outcomes of Ho et al. [43], numerical outcomes of Sheikhzadeh et al. [44], and numerical outcomes of Motlagh and Soltanipour [36] with various Rayleigh numbers for ϕ=0.03; and (**b**) the experimental outcomes of Putra et al. [45] and the numerical outcomes of Corcione et al. [41] with various Rayleigh numbers at ϕ=0.01, N=0, and R=0.

**Figure 5:**
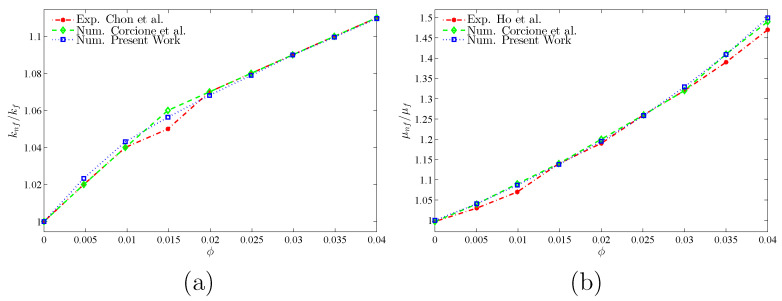
Comparisons of: (**a**) thermal conductivity ratio between the current numerical results, Chon et al. [46] (experimental), and Corcione et al. [41] (numerical); and (**b**) dynamic viscosity ratio of the current results with Ho et al. [43] (experimental) and Corcione et al. [41] (numerical) at $Ra=3.37\times 10^{5}$, N=0, and R=0.

To verify the current numerical data, the outcomes were compared with earlier published numerical outcomes achieved by Costa and Raimundo [13] concerning the problem of mixed convection heat transfer into a cavity filled with pure liquid and heated vertically into the presence of rotating cylinder, as depicted in Figure 2. The streamlines indicate the CW rotation for Ω=−500, 0, and 500. At Ω=500, the streamlines are more powerful than Ω=0 and 500. In addition, the isotherms show horizontal lines for Ω=0 for both works. Besides, comparisons performed among the existing streamlines, isotherms, and nanoparticles volume fraction inside a free cavity and the numerical ones received by Corcione et al. [41] and Wang et al. [42] are demonstrated in Figure 3, where similar nanoparticle distribution is recorded. In addition, for natural convection flow in a cavity filled with nanofluid utilizing Buongiorno’s two-phase model as exhibited in Figure 4a, the average heat transfer matched the experimental results of Ho et al. [43] and numerical outputs of Sheikhzadeh et al. [44] and Motlagh and Soltanipour [36] with various Rayleigh numbers toward ϕ=0.03. Figure 4b displays a comparison among the current outcomes and the experimental arrangements of Putra et al. [45] and the numerical result of Corcione et al. [41] using Buongiorno’s model and for various Rayleigh numbers at ϕ=0.01, N=0, and R=0. Figure 5 gives alternative observations concerning the enhancement in the thermal conductivity and dynamic viscosity due to the addition of the ${\mathrm{Al}}_{2}O_{3}$ nanoparticles with two different experimental outcomes and the numerical outcomes of Corcione et al. [41] as well. The maximum accuracy of the measurements of thermal conductivity in the study of Chon et al. [46] (experimental) was 3.90%. The accuracy of viscosity measurements in the study of Ho et al. [43] (experimental) was 1%. Based on those validations, the numerical results of the actual numerical code significance to a great level of reliability.

## 4. Results and Discussion

Current segment displays numerical outcomes concerning the streamlines, isotherms, and nanoparticle distribution among two cases of the angular rotational velocity (Ω and −Ω), Richardson number (0.01≤Ri≤100), nanoparticle volume fraction (0≤ϕ≤0.04), number of undulations (0≤N≤4), and dimensionless radius of the rotating cylinder (0.05≤R≤0.25), where the values of other parameters are fixed at Re=100, $k_{s}=0.76$, Pr=4.623, $Le=3.5\times 10^{5}$, $Sc=3.55\times 10^{4}$, Θ=360, and $N_{BT}=4.1$. The thermophysical properties of the base liquid and solid Al_2_O_3_ phases are tabulated in Table 1. Following the Buongiorno’s model, the suspension of nanoparticles is assumed a dilute suspension, and, hence, the outcomes could be valid for low volume fractions of nanoparticles, i.e., ϕ<0.05.

**Figure 6:**
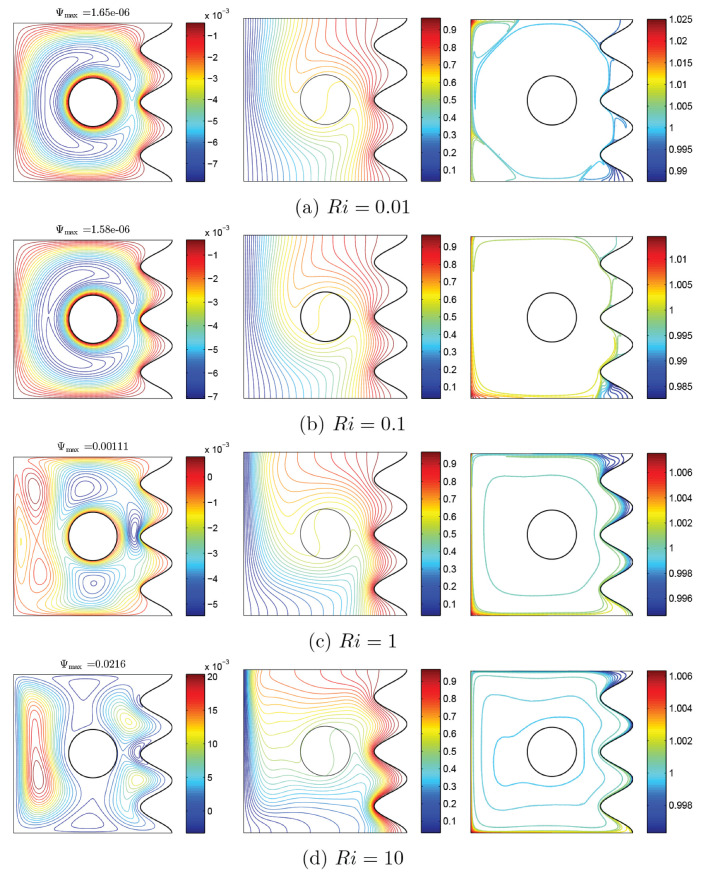
Variations of the (**left**) streamlines, (**middle**) isotherms, and (**right**) nanoparticle distribution evolution by Richardson number (Ri) for Case 1 (Ω), ϕ=0.02, N=3, and R=0.15.

**Figure 7:**
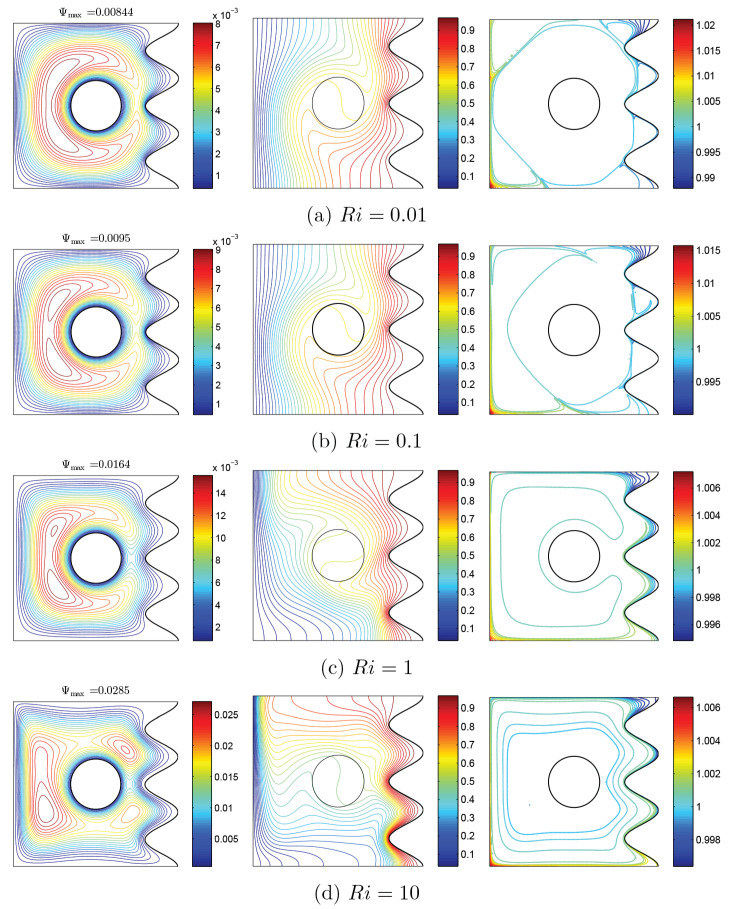
Variations of the (**left**) streamlines, (**middle**) isotherms, and (**right**) nanoparticle distribution evolution by Richardson number (Ri) for Case 2 (−Ω), ϕ=0.02, N=3, and R=0.15.

Figure 6 and Figure 7 show the changes of streamlines and isotherms among the changes of Richardson number (Ri) and also with clockwise and CCW rotations of the rigid body. In these figures, distribution of volume fraction and remarkable changes with the variations of Ri can be observed. In general, considering the momentum equation, the velocity of nanofluid increases with an increase of Ri, which is indicated in maximum amounts of streamlines. Another notable point observed in streamlines in both states is the occurrence of tiny whirlpools created by the increase of Ri. When Re is constant, an increase of Ri leads to increase of Gr, and with the addition of buoyancy, the pattern of flow increase and some whirlpools are observed at high values of Ri. In two states, as Ri increases, isothermal lines change with a certain trend, so that with the increase of Ri, isothermal lines of corrugated wall get closer to each other, as clearly observed at the lower corner of cavity. In the mentioned model, nanoparticles movement is affected by two factors: Brownian and thermophoresis. In the state of the low gradient for Ri, the temperature is very high. Thus, nanoparticles travel from the cool wall toward the warm one, as a result of the temperature gradient. Among the rise of Ri and drop of the gradient of temperature, the movement of nanoparticles will be confined to a couple of lines. However, due to Brownian motion, the movement of nanoparticles near the rotating rigid body is observable; therefore, as shown in Figure 6, the nanoparticles travel from the right wavy surface and middle of the cavity to the bottom and left walls, especially the corners of the left wall. This trend can also be seen for nanoparticle distribution in Figure 7. Furthermore, the thermophoresis force tends to move the nanoparticles in a direction opposite to the temperature gradient. At the hot side of a nanoparticle, the liquid molecules are with more energy, and the impact of collision of nanoparticles and the liquid molecules induces a net force. This net force, thermophoresis force, tends to move the nanoparticles from hot to cold. The Brownian motion tends to make the nanoparticles in the liquid uniform. Hence, the Brownian motion exerts a net force on the nanoparticles to move them from a high concentration area to a low concentration one. This way, the Brownian motion and thermophoresis forces adjust the distribution of nanoparticles in a liquid in the presence of notable temperature gradients.

**Figure 8:**
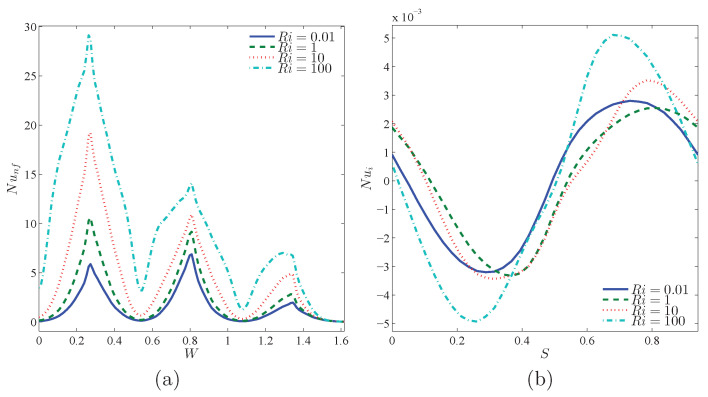
Variations of local Nusselt number interfaces with (**a**) *W* and (**b**) *S* for different Ri at Case 1 (Ω), ϕ=0.02, N=3, and R=0.15.

**Figure 9:**
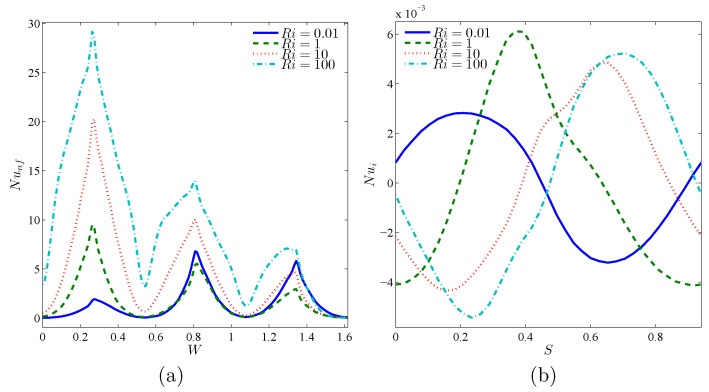
Variations of local Nusselt number interfaces with (**a**) *W* and (**b**) *S* for different Ri at Case 2 (−Ω), ϕ=0.02, N=3, and R=0.15.

Figure 8 reveals the variations of local Nu against the warm wall and rotating rigid body, with an addition of Ri. Since Ri raises, isothermal lines shown in Figure 6, near the corrugated wall, get closer and become more dense and therefore the temperature gradient increases. Local Nu is also expected to increase. The density of isothermal lines at the concave points of the corrugated wall is much higher than at the convex points, and hence the local Nu gets its maximum value at the concave points. By moving along the circumference of the rotating rigid wall, temperature gradient decreases and gets a negative value. This negative gradient firstly increases and then drops. Afterwards, isothermal lines will have a positive slope, and after passing the maximum point, the negative gradient decreases. This trend accounts for the occurrence of a minimum and a maximum location at the local Nu of the rotating rigid body. The same pattern is noticed for local Nu results in Figure 9.

**Figure 10:**
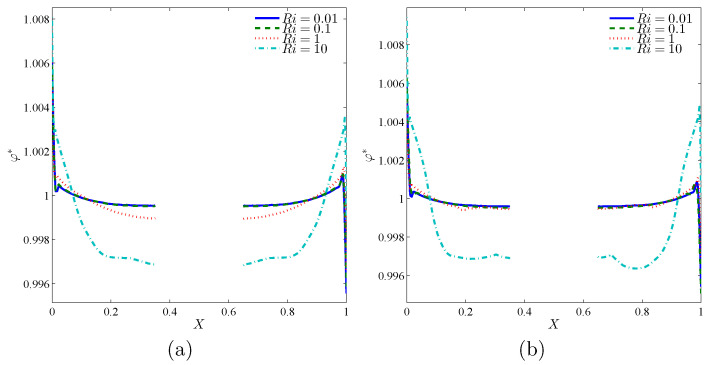
Variations of local normalized solid volume fraction interfaces with the horizontal center line (Y=0.5) for different Ri at (**a**) Case 1 (Ω) and (**b**) Case 2 (−Ω) for ϕ=0.02, N=0, and R=0.15.

Figure 10 shows the distribution of nanoparticles at the middle plane of the cavity (Y=0.5) along the *X*-direction. Figure 10a,b depicts the results for the rotating Cases 1 and 2, respectively. As seen, both cases show similar behavior except the concentrations near the rotating cylinder. In both figures, a sharp variation of particles concentration next to the hot and cold surfaces can be observed. A high level of nanoparticles could be located at the cold surface. That is due to the thermophoresis effect, which tends to move the nanoparticles of hot to cold zones. Close to the heated surface, the concentration of nanoparticles is low. This is again due to the thermophoresis effect, which sweeps the particles away from the hot surface. The intensity of the concentration boundary layer remains minimum compared to the temperature and hydrodynamic boundary layers. This thin boundary layer is the results of the vast Lewis and Schmidt numbers for nanofluids.

Far from the walls, where the temperature gradients are smooth, a uniform concentration of nanoparticles could be found. The consistent level of nanoparticles at such regions is due to the Brownian motion effects, which tend to move the nanoparticles of an enormous concentration area toward a low concentration one. At the center of the cavity, where the rotating disc is located, there are no nanoparticles, and, hence, there is no concentration gradient. In the case of low Richardson number, the influence of the cylinder’s rotation on the concentration distribution is minimal since the rotation of the solid cylinder remains slow compared via the natural convection flow. In the case of high Ri, the concentration profiles are greatly affected by the rotation and shifted downward. This downward shift is due to the change of the cold flow toward the down bottom of the cylinder.

**Figure 11:**
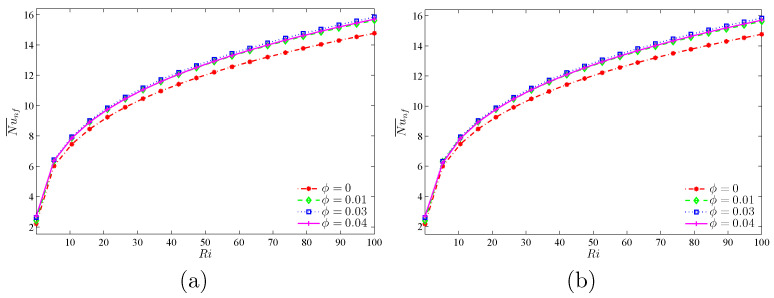
Variations of the average Nusselt number with Ri for different ϕ for (**a**) Case 1 (Ω) and (**b**) Case 2 (−Ω) at N=3 and R=0.15.

**Figure 12:**
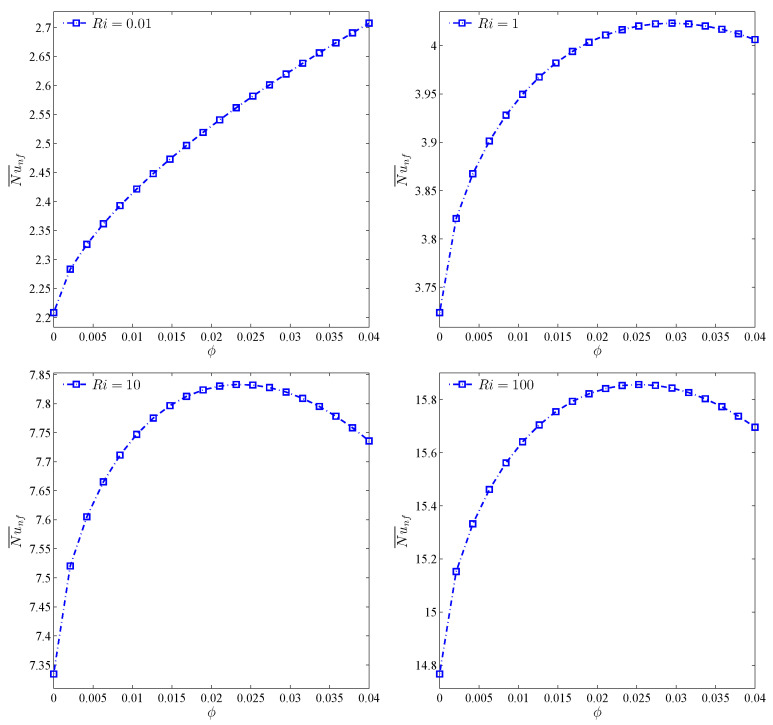
Variations of the average Nusselt number with ϕ for different Ri at Case 1 (Ω), N=3 and R=0.15.

Figure 11 represents the continuous increase of the average Nu, i.e., the heat transfer rate, with an increase of Ri, for both cases. However, the maximum increase of heat transfer is observed at the volume fraction of 3%. To investigate this trend with more analysis at constant amounts of Ri, the effects of variations of volume fraction toward the average Nu was analyzed (Figure 12). For all values of Ri, except for the case of Ri=0.01, an optimum point is observed for the average Nu number. This means that, given a constant Re, Gr increases with the increase of Ri. Thus, forced convection is overshadowed by the free type. In the free convection of nanofluids, an addition of volume fraction along with the rise in viscosity points to the decrease of heat transfer and, hence, for the geometry studied, an increase of volume fraction after passing the maximum score heads to the reduction of Nu. ϕ=0.03 may be considered as the optimum point for volume fraction.

**Figure 13:**
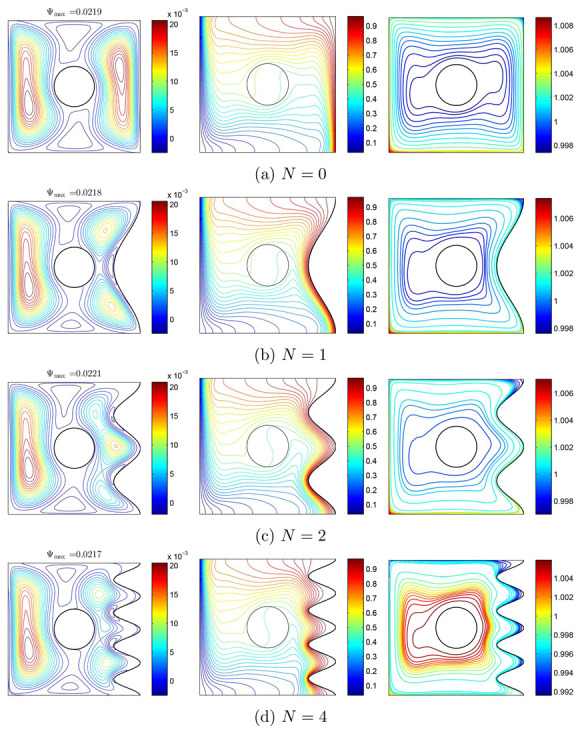
Variations of the (**left**) streamlines, (**middle**) isotherms, and (**right**) nanoparticle distribution evolution by number of undulations (*N*) for Case 1 (Ω), Ri=10, ϕ=0.02, and R=0.15.

**Figure 14:**
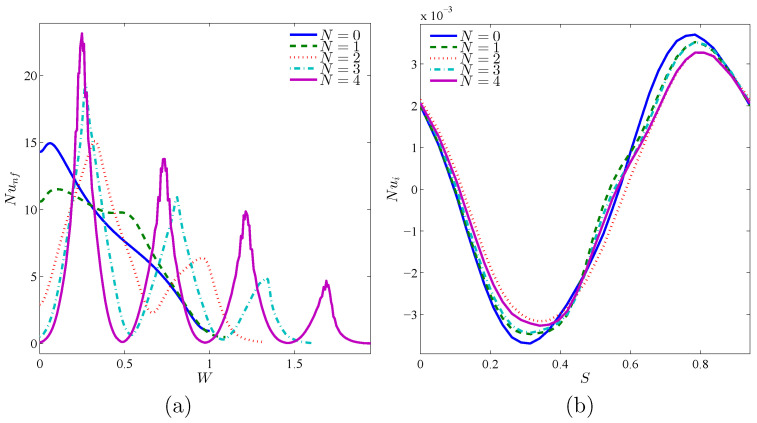
Variations of local Nusselt number interfaces with (**a**) *W* and (**b**) *S* for different *N* at Case 1 (Ω), Ri=10, ϕ=0.02, and R=0.15.

Figure 13 reveals the impact of increasing the number of grooves toward the flow patterns, temperature, and heat transfer. At the two sides regarding the rigid body, streamlines have CCW rotations, so that they are pulled toward the corrugations as the right warm wall gets corrugated. The maximum value of streamline is seen at N=2. Isothermal lines have a remarkable density above the cool wall and below the warm wall, so that the density will be highly noticeable at the concave points as the warm wall becomes corrugated. Primarily, the movement of nanoparticles has a meaningful symmetry around the rigid body, so that with corrugating the warm wall and accordingly the change of flow pattern and temperature, they are pulled into the concave regions of the warm wall (Figure 13). Generally, via increasing *N*, the nanoparticles migration decreases. The concentration of nanoparticles near the rotating cylinder at N=4 is higher than other parts of the cavity. In different values of *N*, the level of nanoparticles at the corner of the left and bottom walls is very notable. Considering the isothermal lines, the maximum value of local Nu of the warm wall is seen at N=4. However, at X=L and Y=0, the maximum value of Nu happens at N=0. As noted above, local Nu has some peaks at the concave points. For local Nu, the rotating rigid body has its minimum and maximum values as a result of its high temperature gradient (Figure 14).

**Figure 15:**
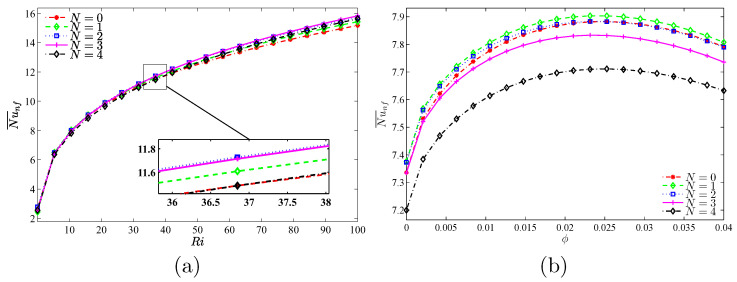
Variations of the average Nusselt number with (**a**) Ri and (**b**) ϕ for different *N* at Case 1 (Ω), R=0.15.

As displayed in Figure 15, the rate of heat transfer increases by the growth of Ri. At a relatively low number of undulations N=1, the highest volume of heat transfer rate and also the maximum increase of it is observed by the rise of volume fraction of nanoparticles. The maximum amount of heat transfer rate is seen at 0.02<ϕ<0.03.

**Figure 16:**
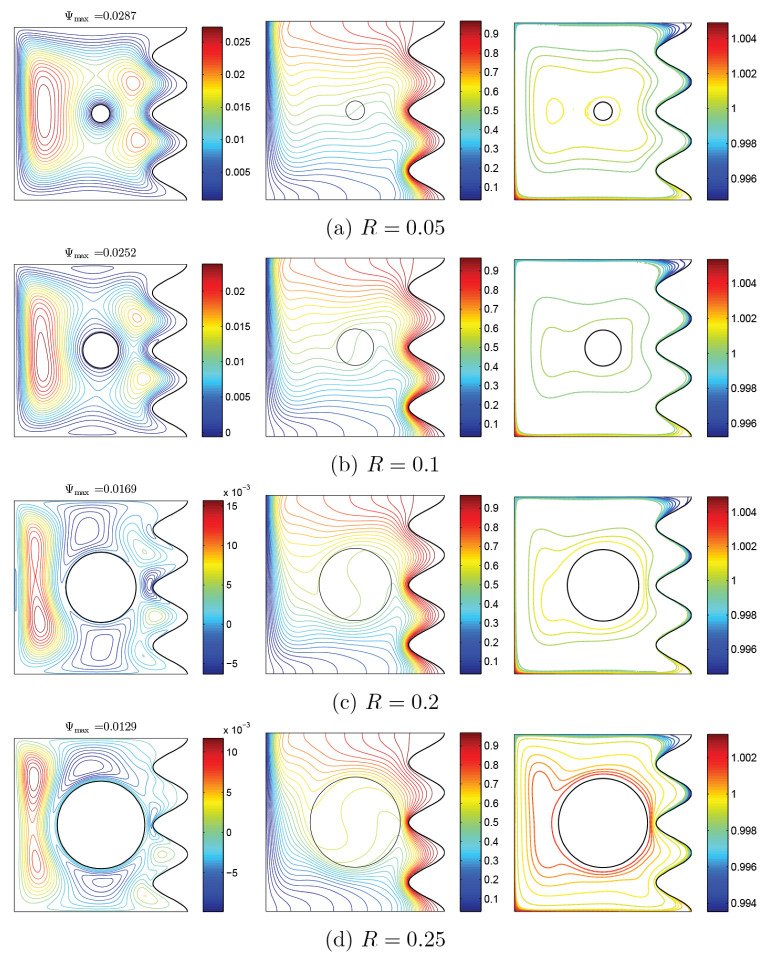
Variations of the (**left**) streamlines, (**middle**) isotherms, and (**right**) nanoparticle distribution evolution by the radius of the rotating cylinder (*R*) for Case 1 (Ω), Ri=10, ϕ=0.02 and N=3.

**Figure 17:**
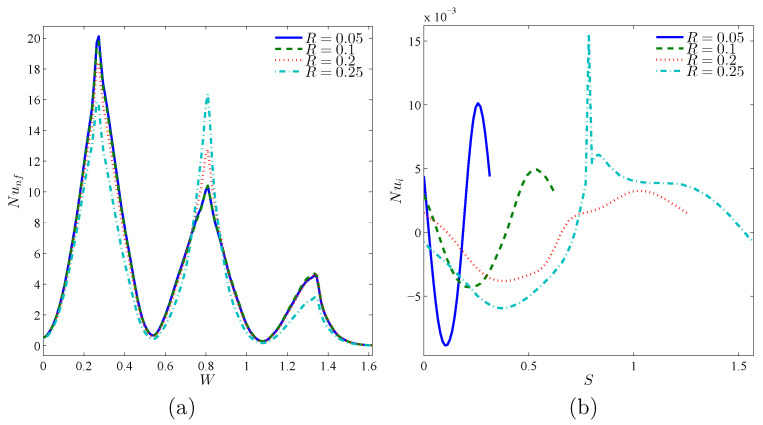
Variations of local Nusselt number interfaces with (**a**) *W* and (**b**) *S* for different *R* at Case 1 (Ω), Ri=10, ϕ=0.02, and N=3.

**Figure 18:**
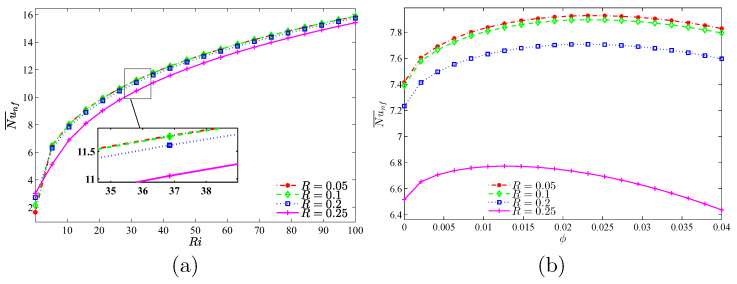
Variations of the average Nusselt number with (**a**) Ri and (**b**) ϕ for different *R* at Case 1 (Ω), N=3.

Figure 16, Figure 17 and Figure 18 show the effects of size of rotating rigid body toward the patterns of flow and heat transfer. Since the volume grows, vortexes get nearer to the body, and the rotations get more prominent because the route of nanofluid has more barriers as the area of rigid body has more cases and thus more massive vortexes are generated. At low values of *R*, the flow pattern makes the isothermal lines become horizontal at a large area of the middle region of cavity. However, with the increase of *R*, isothermal lines get a circle shape at the middle region and are still dense at the concave points of warm wall. For all values of *R*, nanoparticles move from the left corner of the lower surface toward the warm wall, especially the highest point of it. In addition, because of the movement of rigid body and the corroboration of the Brownian motion of nanoparticles, the accumulation of nanoparticles is also noticeable near the moving wall. This accumulation is maximum at R=0.05.

Considering the density of isothermal lines, the maximum amount of local Nu is seen at the first concave region of all amounts of *R*. At the second concave region, considering the proximity of rotating rigid body, the value of local Nu is maximum for large values of *R*, especially for R=0.25. At the last concave region, density of isothermal lines becomes very low and Nu is expected to be lower at the third peak, compared to the other two peaks. This is clearly observed in Figure 17a. At R=0.25, as a result of density at the first two peaks and remarkable reduction of temperature gradient at the last concave region, the behavior of local Nu is highly noticeable. Considering the isothermal lines, Nu of rotating body may be described this way: nearly at the beginning of motion on the wall, temperature gradient is negative, while at the symmetrically opposite region is positive. Thus, a Sin behavior in the Nu of rotating wall, including a negative minimum and a positive maximum, is observed. Considering the aggregation of temperature gradient at R=0.25 and the presence of vortexes, local Nu has a remarkable growth at its positive maximum, in comparison with other values of *R* (Figure 17b).

Considering Figure 18, the maximum value of the average Nu is seen at R=0.05, while for low amounts of Ri, the maximum amount of average Nu occurs at R=0.25. From Ri≥10 and at all values of *R*, the most significant heat transfer is detected at R=0.05. With the increase of ϕ, a different behavior for heat transfer is seen with various values of *R*. For all values of *R*, an optimum value of ϕ is seen and, for various states of *R*, the following values for volume fraction can be offered, which result in the highest amount of heat transfer: 0.05<R<0.2 and ϕ=0.025 as well as R=0.25 and ϕ=0.01.

## 5. Conclusions

Combined convection flow and heat transfer mechanisms toward an enclosed cavity including heated wavy surface and rotating circular cylinder were examined numerically using Buongiorno’s two-phase approach. The current outcomes have directed the following concluding statements:

1. An addition of the average Nu with the rise of Ri, for both CW and CCW rotating, is experienced. In addition, the maximum increase of heat transfer is perceived toward the nanoparticles volume fraction of 3%.
2. The most significant amount of streamline is noticed at a relatively high number of undulations N=2.
3. In the situation of rotating the solid cylinder in counterclockwise (Case 1), the maximum mean Nusselt number is observed at singular undulation N=1 and the nanoparticles volume fraction in the range 0.02<ϕ<0.03.
4. For the case of ruled natural convection (Ri≥10) and all amounts of *R*, the maximum significance of heat transfer is recognized at R=0.05.
5. For all amounts of *R*, an optimum value of ϕ is seen and, for various states of *R*, the following values for volume fraction can be offered, which result in the highest amount of the heat transfer rate: 0.05<R<0.2, ϕ=0.025, and R=0.25, ϕ=0.01.

## Figures and Tables

**Table 1:** Thermophysical properties of base liquid with Al_2_O_3_ nanoparticles at T=310 K [36,47].

Physical Properties	Base Liquid Phase (Water)	Al_2_O_3_
$k\;({\mathrm{Wm}}^{-1}K^{-1})$	0.628	40
$\mu \times 10^{6}\;\left(\mathrm{kg}/\mathrm{ms}\right)$	695	–
$\rho \;(\mathrm{kg}/m^{3})$	993	3970
$C_{p}\;\left(J/\mathrm{kgK}\right)$	4178	765
$\beta \times 10^{5}\;\left(1/K\right)$	36.2	0.85
$d_{p}\;\left(\mathrm{nm}\right)$	0.385	33
